# Pharmacological management of acute myocardial infarction in the municipal district of Rio de Janeiro

**DOI:** 10.1590/S1516-31802001000600003

**Published:** 2001-11-01

**Authors:** Claudia Caminha Escosteguy, Margareth Crisóstomo Portela, Maurício Teixeira Leite de Vasconcellos, Roberto de Andrade Medronho

**Keywords:** Acute myocardial infarction, Healthcare assurance, Quality assurance, Thrombolytic therapy, Infarto agudo do mio cárdio, Avaliação de serviços de saúde, Avaliação de qualidade, Trombólise

## Abstract

**CONTEXT:**

International studies have shown a large variation in the utilization patterns of interventions, in acute myocardial infarction.

**OBJECTIVE:**

To analyze utilization patterns of pharma-cological interventions in acute myocardial infarction and their corresponding effects on hospital mortality.

**DESIGN:**

Cross-sectional study.

**LOCAL:**

Hospitals of the Brazilian National Health System (SUS)in the municipal district of Rio deJaneiro.

**SAMPLE:**

A stratified hospital sample of 391 medical records selected from the 1,936 admissions regis-tered in the SUS Hospital Information System(SIH/ SUS)with a main diagnosis of acute myocardial infarction, in the studied district in 1997.

**MAIN MEASUREMENTS:**

Sex, age, time to treatment, risk factors, severity factors, diagnosis confirmation, use of pharmacological interventions, hospital death, contraindication of the use of thrombolytic therapy, contraindication of aspirin use.

**RESULTS:**

We reviewed 98.2% of the sampled medical records. Acute my ocardial infarction diagnosis was confirmed in 91.7%(95%CI 88.3 to 94.2). 61.5% were men and 38.5%women, with an average age of 60.2 years(SD 2.4). The median time inter-valbetween symptom on set and hospital admission was 11 hours. Hospital mortality was 20.6%(95% CI 16.7 to 25.0). Intravenous thrombolytic therapy was used in 19.5%(95% CI 15.8 to 23.9)of the cases; aspirin in 86.5%(95% CI 82.5 to 89.6); beta-blockers in 49.0%(95% CI 43.8 to 54.1); angiotensin-convertingenzyme(ACE)inhibitors in 63.3%(95% CI 58.2 to 68.1); nitrates in 82.0% (95% CI 82.4 to 89.6); heparin in 81.3%(95% CI 76.9 to 85.0);calcium antagonists in 30.5%(95% CI 26.0 to 35.4). Therewas a significant variation in the use of thrombolytic therapy, beta-blockers, ACE inhibitors, calcium antagonists and heparin among hospitals of different juridical nature.

**CONCLUSIONS:**

There was underutilization of some interventions with well-established efficacy (thrombolytic therapy, aspirin, beta-blockers and intravenous nitrates). The use of calcium antagonists, not supported by scientific evidence in acute myocardial infarction, was quite frequent*. Alogistic model documented the benefit of aspirin, beta-blockers and ACE inhibitor use in reducing thechance ofhospitaldeath.*

## RESUMO

**CONTEXTO:**

Apesar do infarto agudo do miocárdio contar com ampla disponibilidade de evidências científicas para orientar seu tratamento, estudos internacionais têm descrito uma grande variação nos perfis de uso de intervenções, nem sempre com base nessas evidências.

**OBJETIVO:**

Analisar perfis de uso de intervenções farmacológicas no infarto agudo do miocárdio e o efeito correspondente na mortalidade hospitalar no município do Rio de Janeiro.

**TIPO DE ESTUDO:**

Estudo transversal.

**LOCAL:**

Hospitais integrantes do Sistema Único de Saúde (SUS) do município do Rio de Janeiro.

**AMOSTRA:**

Amostra aleatória estratificada por hospital composta de 391 prontuários sorteados a partir do total de 1.936 internações registradas com o diagnóstico principal de infarto agudo do miocárdio no Sistema de Informações Hospitalares (SIH) do SUS em 1997, no município estudado.

**VARIÁVEIS ESTUDADAS:**

Sexo, idade, delta tempo, fatores de risco, fatores de gravidade, confirmação diagnóstica, uso de intervenções farmacológicas durante a hospitalização, óbito hospitalar, contra-indicação ao uso de trombolítico e de ácido acetilsalicílico.

**RESULTADOS:**

Foram revistos 98,2% dos prontuários sorteados; o diagnóstico de infarto agudo do miocárdio foi confirmado em 91,7% dos casos (IC 95% 88,3 a 94,2). 61,5% eram ahomens e 38,5% mulheres, com idade média de 60,2 anos (SD 2,4). A mediana de tempo entre início dos sintomas e hospitalização foi 11 horas. A mortalidade hospitalar foi 20,6% (IC 95% 16,7 a 25,0). Trombólise intravenosa foi usada em 19,5% (IC 95% 15.8 a 23.9) dos casos; ácido acetilsalicílico em 86,5% (IC 95% 82,5 a 89,6); betabloqueadores em 49,0% (IC 95% 43,8 a 54,1); inibidores da enzima conversora do angiotensinogênio (ECA) em 63,3% (IC 95% 58,2 a 68,1); nitratos em 82,0% (IC 95% 82,4 a 89,6); heparina em 81,3% (IC 95% 76,9 a 85,0); bloqueadores de cálcio em 30,5% (IC 95% 26,0 a 35,4). O uso de trombolíticos, betabloqueadores, inibidores da ECA, bloqueadores de cálcio e heparina variou significativamente entre as naturezas jurídicas dos hospitais.

**CONCLUSÕES:**

Houve subutilização de algumas intervenções com eficácia documentada (trombolíticos IV, ácido acetilsalicílico, betabloqueadores e nitratos IV) e uso relativamente disseminado de bloqueadores de cálcio sem base em evidências científicas. Através de modelo logístico documentou-se o efeito benéfico do uso de ácido acetilsalicílico, betabloqueadores e inibidores da ECA sobre o risco de morte hospitalar.

## INTRODUCTION

Cardiovascular diseases (CVD) play a preponderant role in the morbidity-mortality indicators in Brazil, and have been the primary cause of proportional mortality in the country since the 1960's. Coronary heart disease (CHD), including acute myocardial infarction, is the main component of that mortality in the cities of the South and Southeast Regions.^1,2^

Most of the hospitalized cases of acute myocardial infarction in developed countries are treated in coronary care units (CCU). In recent years, the wide availability of randomized clinical trials has facilitated the development by specialist panels of guidelines for evidencebased treatment in cardiology. One of the most-used guidelines was the result of a task force organized by the American College of Cardiology and the American Heart Association, with its latest revision in 1999.^3^ The Brazilian Society of Cardiology has also published national guidelines for the treatment of several cardiological diseases, including those related to coronary heart disease.^4^

However, some studies demonstrate that the existence or even the knowledge of those guidelines has not guaranteed evidence-based medical care. There is a huge variation in therapeutic patterns for acute myocardial infarction reported from around the world, often showing non-adherence to well-established protocols.^5-15^ There is also a huge variation in the hospital mortality observed in different hospitals. This variation may be related, among other reasons, to differences in the severity of the cases, as well as to differences in the quality of medical care.^6^^,^^9-14^^,^^16-20^

So far, we have not found studies on the utilization patterns of pharmacological management for acute myocardial infarction in the municipal district of Rio de Janeiro as a whole. The present study was developed with the objective of analyzing those patterns and their corresponding effects on inpatient mortality in acute myocardial infarction hospitalizations covered by the Brazilian National Health System (SUS) in the Rio de Janeiro municipal district.

## METHODS

The study protocol was approved by the Commission of Ethics in Research of the National Public Health School/FIOCRUZ. Informed consent from the hospital administrations was requested for the data collection. Patients would not be identified and feedback of the results to the participating hospitals was guaranteed. The fieldwork and data collection form are detailed in another publication.^21^

This was a sectional study of a stratified random sample of 391 medical records, selected from 1,936 acute myocardial infarction admissions registered by the SUS Hospital Information System (SIH/SUS), through its Authorization of Hospital Admission (AIH) form, in the municipal district of Rio de Janeiro during 1997. The determination of the sample size was based on the proportions of patients and fatal events, with a relative error of 0.10 and significance level of 0.05, in a sample model stratified by hospital, with proportional allocation without reposition.^22^ The sample was made up of 391 medical records (corresponding to the number of patients), proportionally selected from the 22 hospitals included in the sample. Hospitals that registered less than 10 cases were excluded. Data were collected from the sampled medical records, including variables relating to diagnosis confirmation, risk factors, disease severity and the use of therapeutic interventions.

The diagnosis was confirmed by means of the criteria for definitive or possible acute myocardial infarction adopted by the World Health Organization (WHO), MONICA Project.^23^ Clinical classification of the hemodynamic status of the patients was made according to Killip & Kimball.^24^

To study the effect of the pharmacological interventions on the risk of hospital death we used multivariate analysis with logistic regression. We also tested random effects at the hospital level that were not detected in any of the presented models. Odds ratios (OR) and their respective 95% confidence intervals (95% CI) were estimated. A p-value of 0.05 or less was considered statistically significant. During the analysis, the possibility of confounding by indication was checked through the inclusion in the model of variables related to case severity and the indication of interventions, as proposed by some authors.^25^

## RESULTS

We reviewed 384 patient records corresponding to the sampled hospitalizations (a loss of 1.8%). The diagnosis was confirmed in 91.7% (95% CI = 88.3 to 94.2) of the cases, according to the criteria of definitive or possible acute myocardial infarction.^23^ There were 61.5% men and 38.5% women in the sample (a ratio of 1.6:1), with an average age of 60.2 ± 2.4 years. The median time interval between symptom onset and hospitalization was 11 hours. 81.3% (95% CI = 76.9 to 85.0) of the cases were treated in coronary care units or intensive care units (ICU). Hospital mortality was 20.6% (95% CI = 16.7 to 25.0).

Table 1 shows the frequency of some pharmacological interventions used in the sample. The proportion of records in which information concerning the use of interventions was missing (use unknown) varied from 3.7% for thrombolytic therapy to 8.1% for lidocaine use, and around 6% for the other studied drugs. ACE inhibitors refer to the group of drugs that inhibit the angiotensin-converting enzyme. The proportion of interventions used in the confirmed acute myocardial infarction cases was similar to that shown in Table 1.

**Table 1:** Frequency of some pharmacological interventions in the 384 acute myocardial infarction cases of the sample, Rio de Janeiro municipal district, 1997

*Intervention*	*F*	*Use (reported in the medical record) %*	*95% CI*
***Thrombolytictherapy ^1^***	***75***	***19.5***	***15.8 to 23.9***
***Aspirin***	***332***	***86.5***	***82.5 to 89.6***
***Beta-blockers***	***188***	***49.0***	***43.9 to 54.1***
***Nitrates (oral and/or IV)***	***315***	***82.0***	***77.7to 85.7***
***Heparin (SC and/or IV)***	***312***	***81.3***	***76.9 to 85.0***
***ACE inhibitors***	***243***	***63.3***	***58.2 to 68.1***
***Calcium antagonists***	***117***	***30.5***	***26.0 to 35.4***
***Lidocaine***	***35***	***9.1***	***6.5 to 12.6***

***^1^ 7cases who received thrombolytic therapy before arriving at the admission hospital are included. SC = Subcutaneous;IV=intravenous.***

The thrombolytic agent used was intravenous streptokinase in all the 75 cases treated with pharmacological thrombolysis. Seven of these cases received the thrombolytic treatment in another unit, different from the hospital that generated the AIH form. One of these cases was assisted by the ambulance of the Firemen's Special Rescue Group. Therefore, 1.8% of the acute myocardial infarction cases received thrombolytic treatment before hospital admission. It is worth pointing out that at this time there was an official agreement between the Municipal Hospital Paulino Werneck and the University Hospital Clementino Fraga Filho, for teleconsulting on the evaluation of the thrombolytic indication, thereby favoring earlier treatment at the level of first emergency assistance in the municipal hospital, with later transfer to the university coronary care unit.

Only 72.3% of the 332 patients who received aspirin had the treatment administered from the first day at the hospital.

In Table 1, oral and intravenous (IV) uses are aggregated for both beta-blockers and nitrates. For beta-blockers, intravenous use was exceptional. For nitrates, intravenous use was observed in 18.8% (72 cases) of the sample, versus oral use in 79.2% (304 cases). 25.5% (98) of the acute myocardial infarction cases received intravenous heparin, versus 71.9% (276) of subcutaneous (SC) heparin. In 66 cases (17.2% of the sample), both intravenous and subcutaneous heparin were used during the hospitalization.

Other pharmacological interventions frequently observed in the sample were: H_2_ -receptor blockers - 53.7%; diuretics - 22.9%; inotropic agents (dobutamine, dopamine) -14.1%; digitalis - 10.4%; amiodarone - 7.3%; oral anticoagulants - 5.7%; atropine – 3.4%; sodium nitroprusside - 1.3%.

Table 2 shows a significant variation in the use of thrombolytic therapy, ACE inhibitors and heparin among hospitals of different juridical nature. Although, in general, the variation observed in the use of betablockers and calcium antagonists was not statistically significant, there was significant variation in their use between some hospitals of different juridical nature. There was no statistically significant variation in the use of aspirin and nitrates.

**Table 2:** Variation in the use of some pharmacological interventions according to the juridical nature of the hospitals in the acute myocardial infarction sample, Rio de Janeiro municipal district, 1997

*Intervention*	*State*		*Federal*		*Municipal*		*Private*		*University*		*^p^*
	*f*	*%*	*f*	*%*	*f*	*%*	*f*	*%*	*f*	*%*	
***Aspirin***	***72***	***84.7***	***98***	***84.5***	***126***	***90.0***	***10***	***83.3***	***26***	***83.9***	***NS***
***Thrombolyticagents***	***4***	***4.7***	***24***	***20.7***	***41***	***29.3***	***0***	***0***	***19.4***	***15***	***0.001***
***Beta-blockers***	***42***	***49.4***	***59***	***50.9***	***64***	***45.7***	***3***	***25.0***	***20***	***64.5***	***0.10***
***ACE inhibitors***	***50***	***58.8***	***77***	***66.4***	***100***	***71.4***	***0***	***0***	***16***	***51.6***	***0.001***
***Nitrates (oral/IV)***	***72***	***84.7***	***93***	***80.2***	***117***	***83.6***	***9***	***75***	***24***	***77.4***	***NS***
***Calcium antagonists***	***31***	***36.5***	***33***	***28.5***	***45***	***32.1***	***5***	***41.7***	***3***	***9.7***	***0.10***
***Heparin (SC/IV)***	***66***	***77.7***	***97***	***83.6***	***122***	***87.1***	***4***	***33.3***	***23***	***74.2***	***0.001***
***Lidocaine***	***8***	***9.4***	***11***	***9.5***	***12***	***8.6***	***0***	***0***	***4***	***12.9***	***NS***

***Total number of cases by juridical nature of the hospital:state - 85;federal- 116;municipal- 140;private- 12;university-31. SC = Subcutaneous;IV=intravenous.***

The frequency of beta-blocker use was lowest in private hospitals and highest in university hospitals; this difference was significant (P = 0.02); the difference between federal and private hospitals reached a p value equal to 0.09; the difference between other juridical natures was not significant. Calcium antagonists use was highest in the private hospital and lowest in the university hospitals (P = 0.05). The p value for the comparison between university and federal hospitals was 0.03; between private and state hospitals it was 0.10; and between private and municipal hospitals it was not significant.

Lidocaine use was very similar among the state, federal and municipal hospitals, and a little higher in the university hospitals; the use of this anti-arrhythmic agent was not reported in the private hospital.

The municipal hospitals presented the highest frequency of thrombolytic agents, ACE inhibitors and heparin use. Aspirin use was also higher in the municipal hospitals, although without statistical significance. The university hospitals had the highest frequency of beta-blocker use and the lowest of calcium antagonists. Although in this sample private juridical nature was represented by only one hospital, it is relevant to note that not even one patient received thrombolytic therapy or ACE inhibitors, and that the use of betablockers and heparin was the lowest in the sample. On the other hand, the use of calcium antagonists, not supported by scientific evidence of efficacy in acute myocardial infarction, was highest in this private hospital.

Table 2 aggregates intravenous and subcutaneous use of heparin. Subcutaneous use was more frequent (71.9%) and associated with lower mortality (13%) than for intravenous use (frequency = 25.5% and mortality = 20.4%). The variation according to the juridical nature of the hospitals was significant for both heparin types (P < 0.005). The most frequent use of subcutaneous heparin occurred in the municipal hospitals (78.6%), followed by federal (75%), state (69.4%), university (51.6%) and private (33.3%) ones. Concerning the intravenous use of heparin, the university hospitals showed the highest frequency (45.2%), followed by state (25.9%), federal (25.9%) and municipal (22.9%) hospitals. Intravenous use of heparin was not reported in the private hospital.

We studied the factors associated with the variation in the use of some interventions through logistic regression modeling. Factors significantly associated with the variation in the chance of thrombolytic agent use are shown in Table 3. All the studied variables are categorical; the reference category is not explicit in the Table and it is complementary to the explicit one.

**Table 3:** Factors associated with the use of thrombolytic therapy in the acute myocardial infarction sample, Rio de Janeiro municipal district, 1997, logistic model

*Variable*	*Parameter*	*Standard error*	*Pr >* χ*^2^****P***	*OR*	*95*	*CI%*
***Intercept***	***-3.807***	***0.779***	***0.0001***	***-***	***-***	
***FemaleSex***	***-0.737***	***0.357***	***0.04***	***0.48***	***0.24 to 0.96***	
***Symptom onset***≤ ***6 hours***	***2.593***	***0.328***	***0.0001***	***13.37***	***7.03 to 25.40***	
***Coronary care units or ICU admission***	***1.759***	***0.773***	***0.02***	***5.81***	***1.28 to 26.41***	
***Juridical nature of the hospital Municipal hospital***	***0.836***	***0.319***	***0.009***	***2.31***	***1.23 to 4.31***	

***Concordance = 81.4%.*** χ***^2^ (covariates) = 109.683 with 5 DF (P = 0.0001). ICU - Intensive care Units.***

There was a non-significant trend of smaller chance for thrombolytic therapy use associated with an age of 75 years or older (OR = 0.68; 95% CI = 0.20 to 2.26). The observed significant positive associations with thrombolytic therapy use were time to treatment (delta time) equal to six hours or less, coronary care unit or intensive care unit admission and municipal hospitals (in relation to any other juridical nature).

Female sex was associated with a smaller chance of thrombolytic therapy use, even when the model was saturated with other variables such as age, diabetes, previous history of acute myocardial infarction and systemic hypertension. It is worth noting that those variables did not reach statistical significance and their exclusion did not modify the model in a substantial way.

**Figure 1:**
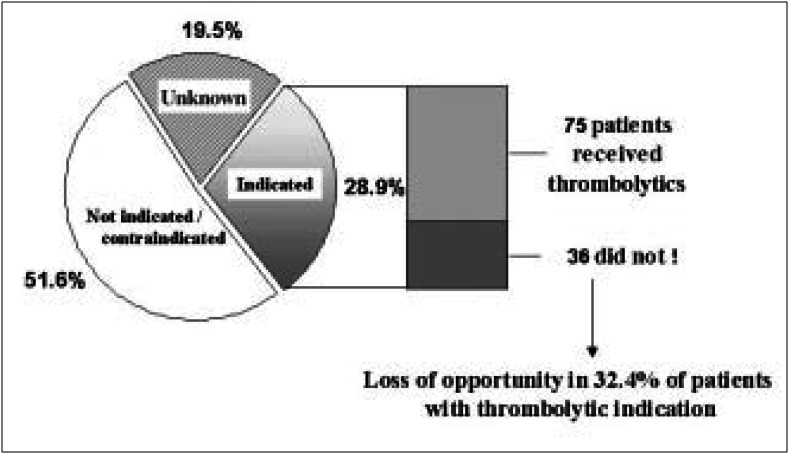
Thrombolytic therapy: frequency of indicated use versus use not indicated in the acute myocardial infarction sample, Rio de Janeiro municipal district, 1997.

Figure 1 shows the distribution of the cases according to the presence of contraindication to thrombolytic therapy, as reported in the medical record.

111 cases in the sample (28.9%) had thrombolytic therapy indication and 198 (51.6%) had at least one explicit contraindication to this therapy (or did not have indication) in the medical record. There was a high frequency of unknown indication or contraindication (19.5% or 75 cases) and it tended to be greater in women (24.3% versus 16.5%; P = 0.06).

The cases with contraindication to thrombolytic therapy included those where delta time, baseline 12-lead electrocardiogram (ECG) or clinical history were not compatible, and those with clear contraindications to thrombolysis, according to evidence-based guidelines.^3^ In the sample, there was at least one contraindication report for thrombolytic therapy in 58.1% of the women and 47.5% of the men (OR = 2.51 with 95% CI = 1.45 to 4.38; P = 0.0004). This fact could at least partially explain the smaller chance of thrombolytic therapy in women, as shown in Table 3.

Overall, 198 cases (51.6%) of the sample presented at least one contraindication (or nonindication) for thrombolytic therapy; 20 cases presented two or more contraindications. Table 4 describes the reported causes of contraindications. The distribution was similar between both sexes, except for a non-significant trend of a higher frequency of previous history of stroke in women (6.8%) than in men (3.8%).

**Table 4:** Causes of contraindication for the use of thrombolytic therapy in the 384 cases in the acute myocardial infarction sample, Rio de Janeiro municipal district, 1997

*Causes^1^*	*f*	*%*
***Symptom onset(time totreatment) > 12 hours***	***107***	***27.9***
***Electrocardiogram (ECG)without indication***	***63***	***16.4***
***Previous history of stroke***	***19***	***5.0***
***Unfavorable risk-benefit ratio***	***9***	***2.3***
***Recent large surgery^1^***	***6***	***1.6***
***Other contraindications (miscellaneous)***	***14***	***2.7***
***Unknown (not related in the medical record)***	***75***	***19.5***
***Total number of cases with at least one cause^2^***	***198***	***51.6***

***^1^ 2 cases of by pass surgery. ^2^ Total number of cases with more than one cause = 20.***

The term "unfavorable risk-benefit ratio" included cases where there was an explicit discussion in the medical record concerning the careful weighing up between relative contraindications and supposed benefit of thrombolysis for that individual patient. This discussion usually involved age over 80 years, time to treatment around 12 hours, absence of pain or ECG without ST segment elevation and already having the presence of Q wave.

In Table 4, other contraindication causes were five cases with undetermined delta time, four cases of severe systemic hypertension, two cases of suspected initial aortic dissection, one case of cardiac arrest in another hospital with a prolonged and traumatic resuscitation, one case of recent digestive hemorrhage and one case of a peptic duodenal ulcer.

Seventy-five of the 111 cases of the sample with explicit indication of thrombolytic therapy were indeed submitted to this treatment. Therefore, 36 cases that were eligible for thrombolysis did not receive the treatment, representing a loss of opportunity in getting reperfusion therapy in 32.4% of the cases with explicit indication according to the medical record.

Multivariate analysis identified the following factors associated with a greater chance of aspirin use: acute myocardial infarction without Q wave (OR 4.83; 95% CI, 1.02 to 22.77) in relation to any other ECG pattern; beta-blocker use (OR 11.24; 95% CI 3.70 to 34.13) and ACE inhibitor use (OR 4.11; 95% CI 1.89 to 8.94), in relation to the non-use of each one of these drugs. The level of concordance for this logistic regression model was 83.5%. Aspirin was the intervention with the best adherence among the hospitals, showing one of the most uniform utilization patterns in the sample.

There was an explicit report of contraindication for aspirin in nine cases (2.3%). Of these, seven were reports at the time of hospital admission, justifying the non-introduction of the treatment (peptic ulcer/gastritis - 4; chronic use of oral anticoagulants - 1; postoperative - 2), and two were reports during the hospitalization, justifying suspension of the treatment (epigastric pain - 1; digestive hemorrhage- 1). Therefore, only seven of the 29 cases in the sample that did not receive aspirin presented a clear contraindication report.

Multivariate analysis showed the following factors associated with a greater chance of betablocker use: younger age group (up to 60 years) with OR 1.91 (95% CI 1.21 to 3.01) in relation to older age group; Killip class I on admission with OR 4.00 (95% CI 1.82 to 8.79) versus Killip > I; anterior wall acute myocardial infarction with OR 1.67 (95% CI 1.04 to 2.69) versus any other location. The presence of diabetes was associated with a smaller chance of beta-blocker use (OR 0.59; 95% CI 0.35 to 0.99) in relation to non-diabetes. The logistic model for beta-blocker use had a concordance level of 75.9%

A greater chance of ACE inhibitor use was observed in association with anterior wall acute myocardial infarction (OR 2.92; 95% CI 1.78 to 4.79) versus any other location; presence of systemic arterial hypertension (OR 2.04; 95% CI 1.27 to 3.27) versus absence; presence of diabetes (OR 1.97; 95% CI 1.10 to 3.53) versus absence; and Killip class II or III on admission (OR 7.13; 95% CI 1.61 to 31.39) in relation to Killip I. There was a trend toward a smaller chance of ACE inhibitor use in Killip class IV on admission (OR 0.36; 95% CI 0.08 to 1.62) versus Killip I.

A greater chance of calcium antagonist use was observed associated with an ECG pattern of acute myocardial infarction without Q wave (OR 2.56; 95% CI 1.42 to 4.61) in relation to any other pattern and with the presence of recurrent ischemia (OR 1.97; 95% CI 1.11 to 3.47) in relation to the absence. There was a trend, although not significant, of lesser use associated with Killip class III or IV on admission (OR 0.24; 95% CI 0.05 to 1.07). However, the factor with greatest impact on the variation in calcium antagonist use in the sample was the juridical nature of the hospital: university hospitals presented the smallest chance of use among all the hospitals. The logistic model for calcium antagonist use had a concordance level of 60%.

Table 5 shows hospital mortality associated with the use (or non-use) of the pharmacological interventions studied in the sample.

**Table 5:** Hospital mortality^1^ in the acute myocardial infarction sample according to the use of some pharmacological interventions

*Drug*	*Used*	*Not used*	*Not used or unknown*	*Used vs. not used*		*Used vs. not used or unknown*	
				*OR (95% CI)*	*^p^*	*OR (95% CI)*	*^p^*
***Thrombolytic therapy***	***13.3***	***20.3***	***22.3***	***0.60 (0.27 to 1.30)***	***NS***	***0.54 (0.24 to 1.15)***	***NS***
***Aspirin***	***14.2***	***69***	***61.5***	***0.07 (0.03 to 0.18)***	***0.000000***	***0.10 (0.05 to 0.20)***	***0.000000***
***Beta-blockers***	***8.0***	***30.8***	***32.7***	***0.19 (0.10 to 0.38)***	***0.000000***	***0.18 (0.09 to 0.34)***	***0.000000***
***ACE inhibitors***	***14.8***	***27.6***	***30.5***	***0.46 (0.26 to 0.81)***	***0.004***	***0.40 (0.23 to 0.68)***	***0.0002***
***Nitrates (oral/ IV)***	***14.9***	***45.7***	***46.4***	***0.21 (0.10 to 0.42)***	***0.000001***	***0.20 (0.11 to 0.37)***	***0.000000***
***Heparin (SC/IV)***	***16.4***	***36.7***	***38.9***	***0.34 (0.17 to 0.68)***	***0.0007***	***0.31 (0.17 to 0.56)***	***0.00002***
***Calcium antagonists***	***14.5***	***20.4***	***23.2***	***0.66 (0.35 to 1.26)***	***NS***	***0.56 (0.30 to 1.05)***	***NS***
***Lidocaine***	***34.3***	***16.4***	***19.2***	***2.67 (1.17 to 6.04)***	***0.009***	***2.20 (0.97 to 4.90)***	***0.04***

***^1^ In percentages(%). SC=Subcutaneous;IV=intravenous.***

Although the frequency of cases of missing information ("unknown" category) concerning the use of those interventions was small (Table 3), we systematically observed high mortality associated with them, generally similar to the mortality associated with non-use of the interventions. However, Table 5 shows that the inclusion of the missing information cases in the reference category for non-use of the intervention generally did not modify the estimated odds ratios in a substantial way. It is also plausible that the cases without explicit negative information in the medical record concerning the utilization of a certain intervention really did not receive it. It is also worth noting that the patients with very precarious information about pharmacological treatment usually died sooner than the other patients did. The average length of stay until death for the eight patients with totally unknown pharmacological therapy was 4.5 days (SD 6.9), with a median of one day and a 75^th^ percentile of 4.5 days. The average length of stay for the other 71 patients who died tended to be longer (8.0 days, SD 9.0), with a median of 4 days and a 75^th^ percentile of 14 days, although the variance analysis did not show a statistically significant difference.

We observed that the mortality associated with the use of intravenous nitrate was higher (18.1%) than that associated with oral use (14.8%), although not significantly. The odds ratio for death associated with the intravenous use of nitrate in relation to non-use was 0.95, with 95% CI 0.46 to 1.95, and the p value was not significant.

The mortality associated with the intravenous use of heparin was higher (20.4%) than that for subcutaneous use (13%), with OR 1.71 (95% CI 0.89 to 3.25, P = 0.08). Subcutaneous use of heparin had a significant association with a smaller chance of death (OR 0.23 with 95% CI 0.13 to 0.42; P < 0.0001) in relation to the non-use.

Lidocaine was associated with an increased chance of death in bivariate analysis.

In a logistic regression model that checked severity (including Killip class on admission, age, sex, ECG pattern), complications, comorbidity (including diabetes), access to coronary care unit and use of invasive interventions, the pharmacological interventions that presented a significant association with smaller chance of death were: use of aspirin (OR 0.30 with 95% CI 0.12 to 0.79, P = 0.01), beta blockers (OR 0.31 with 95% CI 0.14 to 0.70, P = 0.005) and ACE inhibitors (OR 0.44 with 95% CI 0.20 to 0.95, P = 0.04). Thrombolytic therapy did not show significant association in the sample according to bivariate analysis; this fact may be related to the sample size. The concordance level of this model was 90.1%; we did not detect random effect at the hospital level.

## DISCUSSION

There was a huge variation in the use of pharmacological interventions in the sample, except for aspirin and nitrates. These two were the most frequently used interventions and with the most homogeneous pattern among the different juridical natures of the hospitals. Although the use of aspirin was quite common (86.5%), it proved to be less than expected, considering the almost universal indication in acute myocardial infarction (nearly 100%), extremely easy administration, low cost and high efficacy.^3^^,^^26-29^ The underutilization of aspirin has also been reported in other studies.^5,8^

The use of thrombolytic therapy was quite low (19.5% of the cases) and it varied significantly in the sample (Table 2), but always below the pattern of 40% estimated for Brazil by Krauss Silva et al.^28^ The municipal hospitals, on which a good part of the city's emergencies are concentrated, showed the highest frequency of thrombolysis (29.3%). Loss of the opportunity for thrombolytic therapy when indicated has also been reported in another study: the National Registry of Myocardial Infarction-2 revealed that, among the patients with clear indication for thrombolysis, 24% did not receive the therapy.^29,30^ The verification of a smaller chance of thrombolysis use among women has also been reported before,^30^ thereby deserving more detailed studies. We did not find any other association with sex among the use of pharmacological interventions in the sample.

The use of nitrate was mainly via the oral route, whereas the largest described benefits are associated with intravenous use.^3^ The use of intravenous nitrate in only 18.8% of the cases was well below the pattern of 47% estimated for Brazil by another study.^28^ On the other hand, oral use was very frequent, whereas it is generally accepted that nitrates are not indicated for all patients with acute myocardial infarction.^3,4^

The use of beta-blockers was almost entirely restricted to the oral route, whereas studies of the acute phase of infarction were mainly undertaken using the intravenous route. The frequency of beta-blocker use has been observed to vary from 37% to 78% in studies that often did not specify the administration route.^8,11-15^ The estimated incidence of the intravenous use of betablockers in Brazil has been reported as 35% of all acute myocardial infarction cases.^28^

The frequency of ACE inhibitor use was high (63.3%), in a similar way to that reported from a North American register of acute myocardial infarction cases (59.3%)^7^ and higher than the 43% reported in Switzerland.^15^ Among the studied interventions, the incorporation of ACE inhibitors is the most recent one in Brazil. However, these drugs have attained greater adherence among treatment services than have other ones available that were incorporated in the country earlier. The variation in their use was significant among the hospitals; the municipal hospitals had the highest frequency (71.4%, as shown in Table 2).

Generally, the use of calcium antagonists, which is not supported by scientific evidence in acute myocardial infarction, was frequent.

The pattern of beta-blocker and calcium antagonist use in the university hospitals suggests greater adherence of those hospitals to treatment protocols based on scientific evidence.

The possibility of the existence of internal correlation at the hospital level was investigated through the random effects model. Although the variation observed in the use of interventions suggested the existence of intra-class correlation at the level of hospital units, the statistical tests used in the modeling process did not detect any significant random effect. This may be related to the sample size, not only because of the number of superior-level observations (the hospitals), but also because of the number of first-level observations (the cases).

The great variation observed in the use of technologies with well-established indications and efficacy has been the object of analysis by other international studies.^5,7,8^ Drugs with scientific evidence of efficacy in acute myocardial infarction, such as thrombolytic agents, aspirin and beta-blockers, have been underused. On the other hand, some therapies without documented benefit, such as calcium antagonists, have been used relatively often. According to those studies, one of the main explanations for this variation is not the variation of the intervention indication, but rather the different levels of adherence to scientific evidence-based protocols, or even knowledge of them.

In this sample from the municipal district of Rio de Janeiro, the logistic model for the risk of hospital death showed that the use of aspirin, beta-blockers and ACE inhibitors was associated with a reduction in the odds of death. Confounding by indication was checked through the inclusion in the model of variables related to the case severity and intervention indication.^25^

In a general way, the mortality of 20.6% brought out from the sample is far above the low figures that are reached in randomized clinical trials^14^ or selected centers,^6,12^ or estimated from the projection of the efficacy of available technologies.^28^ However, other international studies have also reported such high hospital mortality in acute myocardial infarction, especially when considering multicentric registers of non-selected cases. The USA Second National Registry of Myocardial Infarction (1994 to 1998) reported a 19.7% mortality; when the analysis excluded cases that were transferred from or to other hospitals, the mortality fell to 13.6%.^17^

A multicentric Norwegian study showed a mortality of 18% in 1999;^9^ a multicentric Irish study found 18% in the period 1992-1994;^10^ the multicentric English study in Nottingham, 21.7% in 1992.^11^ Among the Brazilian studies of non-selected cases, there are two that use AIH, describing a mortality of 17.1% in the state of São Paulo in 1997^16^ and 18.4% in the state of Rio de Janeiro in 1995.^20^ Other national studies have described lower mortality, like the one by Passos et al,^12^ which found 12.9% in Salvador, in the period 1993-1994; however, this study selected the participating hospitals according to quality of information and care.

The sex structure and age group of the sample were similar to those in other national studies^12,20,28^ and they did not vary in the sample. The severity of the cases was well described in another study,^22^ and it did not seem worse than that of other series. Therefore, it seems pertinent to investigate the weighting of the care process with regard to the observed deaths.

An Australian study has investigated the possible effects of a quality intervention program for the improvement of quality of acute myocardial infarction hospital care; the program included dissemination of clinical guidelines and clinical indicator feedback to the participating centers.^6^ The study involved 649 patients, from 1996 to 1998, and observed, among other indicators, an increase in the use of thrombolytic therapy from 31% to 70% of cases, a decrease in the hospital mortality from 15.8% to 8.6% and a decrease in the average length of stay from 7.4 to 6.3 days (all with P < 0.05). Those data suggest that combining clinical guidelines with monitoring and close discussion of clinical indicators with treatment services can be useful in improving the quality of care.

## CONCLUSIONS

With the exception of aspirin and nitrates, the use of the studied therapeutic interventions varied significantly among hospitals of different juridical nature in this sample. Among the pharmacological therapies with proven efficacy, the use of aspirin had the largest adherence, although still below the expected when we consider its almost universal indication in acute myocardial infarction. The intravenous use of thrombolytic agents, beta-blockers and nitrates was very reduced. The adherence to the oral use of beta-blockers and nitrates was greater. On the other hand, the use of calcium antagonists, not supported by scientific evidence of efficacy, was relatively widely disseminated.

The multivariate analysis showed a significant beneficial effect associated with the use of aspirin, beta-blockers and ACE inhibitors, confirming their effectiveness in reducing the chance of hospital death.

The documented underutilization of technologies with proven efficacy, including aspirin and beta-blockers, whose beneficial effect on the chance of hospital death was also endorsed in the sample, suggests that greater benefits could be expected from acute myocardial infarction hospital care in the municipal district of Rio de Janeiro, in terms of reducing hospital mortality.
